# Comparison of Autologous and Allogeneic Adipose-Derived Stem Cells in Kidney Transplantation: Immunological Considerations and Therapeutic Efficacy

**DOI:** 10.3390/jcm13195763

**Published:** 2024-09-27

**Authors:** Ljiljana Fodor Duric, Nikolina Basic Jukic, Bozidar Vujicic

**Affiliations:** 1 Medicol Polyclinic, School of Medicine, Croatian Catholic Unoversity, 10000 Zagreb, Croatia; 2 Department of Nephrology, Dialysis and Kidney Transplantation, Clinical Hospital Center Zagreb, Faculty of Medicine, University of Zagreb, 10000 Zagreb, Croatia; nina_basic@net.hr; 3 Department of Nephrology, Dialysis and Kidney Transplantation, Clinical Hospital Center Rijeka, Faculty of Medicine, University of Rijeka, 51000 Rijeka, Croatia; vujicic.bozidar@gmail.com

**Keywords:** mesenchymal stem cells, kidney transplantation, adipose-derived stem cells, regenerative medicine, reperfusion injury

## Abstract

Regenerative medicine shows significant potential in treating kidney diseases through the application of various types of stem and progenitor cells, including mesenchymal stem cells (MSCs), renal stem/progenitor cells, embryonic stem cells (ESCs), and induced pluripotent stem cells (iPSCs). Stem cells possess the unique ability to repair injured organs and improve impaired functions, making them a key element in the research of therapies for kidney tissue repair and organ regeneration. In kidney transplantation, reperfusion injury can cause tissue destruction, leading to an initially low glomerular filtration rate and long-term impact on function by creating irreversible interstitial fibrosis. MSCs have proven useful in repairing early tissue injury in animal models of kidney, lung, heart, and intestine transplantation. The use of stem cell therapies in solid organ transplantation raises the question of whether autologous or allogeneic cells should be preferred. Adipose-derived stem cells (ASCs), characterized by the lack of HLA Class II molecules and low expression of HLA Class I and co-stimulatory signals, are considered immune-privileged. However, the actual risk of graft rejection associated with allogeneic ASCs remains unclear. It has been demonstrated that donor-derived ASCs can promote the development of Treg cells in vitro, and some degree of tolerance induction has been observed in vivo. Nevertheless, a study comparing the efficacy of autologous and allogeneic ASCs in a rat model with a total MHC mismatch for kidney transplantation showed that donor-derived administration of ASCs did not improve the grafts’ survival and was associated with increased mortality through an immunologically mediated mechanism. Given the lack of data, autologous ASCs appear to be a safer option in this research context. The aim of this review was to examine the differences between autologous and allogeneic ASCs in the context of their application in kidney transplantation therapies, considering potential immune reactions and therapeutic efficacy. Some have argued that ASCs harvested from end-stage renal disease (ESRD) patients may have lower regenerative potential due to the toxic effects of uremia, potentially limiting their use in transplantation settings. However, evidence suggests that the beneficial properties of ASCs are not affected by uremia or dialysis. Indeed, some investigators have demonstrated that ASCs harvested from chronic kidney disease (CKD) patients exhibit normal characteristics and function, maintaining consistent proliferative capacity and genetic stability over time, even after prolonged exposure to uremic serum Furthermore, no differences were observed in the response of ASCs to immune activation or their inhibitory effect on the proliferation of alloantigen-activated peripheral blood mononuclear cells between patients with normal or impaired renal function. This review presents the current achievements in stem cell research aimed at treating kidney diseases, highlighting significant progress and ongoing efforts in the development of stem cell-based therapies. Despite the encouraging results, further research is needed to overcome the current limitations and fully realize the potential of these innovative treatments. Advances in this field are crucial for developing effective therapies that can address the complex challenges associated with kidney damage and failure.

## 1. Introduction

Chronic kidney disease (CKD) is one of the cardinal public health problems affecting 10–15% of the general population and causing premature death. It often develops as a result of ongoing kidney injury and scarring from common conditions such as hypertension, diabetes, or nephrolithiasis. Less commonly, it can result from chronic glomerulonephritis or other diseases [1]. Patients with chronic kidney disease have endothelial dysfunction with progressive atherosclerosis and, consequently, different cardiovascular complications, which are the leading cause of death in end-stage kidney disease (ESKD) [2].

There are limited pharmacological strategies available to prevent or alleviate chronic renal failure. These strategies include managing cardiovascular risk factors, avoiding potential renal toxins, and providing causal treatment for acute kidney injury when possible. However, these options often have varying levels of success and come with frequent complications. Despite advancements in other medical fields, the lack of effective therapeutic options for chronic kidney disease (CKD) poses a significant challenge, causing frustration for both patients and healthcare providers. Furthermore, the shortage of organs and complications associated with kidney transplantation necessitates the development of new and innovative therapies. Stem cell-based therapies offer hope for a breakthrough in treating kidney diseases [1]. Given the limitations of current therapeutic approaches in treating kidney diseases and transplantation, exploring new strategies that enhance the regenerative capabilities of stem cells—either through the administration of ex vivo expanded stem cells or by promoting the expansion and differentiation of local progenitor/stem cell populations—represents a significant advancement in research and may open new avenues for improving therapies in these areas. Thus, exploring therapeutic strategies that enhance the regenerative capabilities of stem cells through the administration of stem cells expanded ex vivo or by promoting the expansion and differentiation of local progenitor/stem cell populations represents a promising frontier for future research, both in kidney diseases and kidney transplantation.

## 2. Mesenchymal Stem Cells

### 2.1. Types of Stem Cells

Stem cells can uniquely replicate and differentiate into specialized organ cells, allowing tissues to regenerate and survive most injuries [1,3]. There are four types of stem cells, defined according to their differentiation potential. During the embryonic period, the early stages of development occur due to the unlimited abilities of totipotent zygotic cells, which are later replaced by pluripotent embryonic cells. These pluripotent cells can differentiate into cells of all three germ layers but no longer have the ability to differentiate into placental cells [4].

Pluripotent cells resembling embryonic stem cells can be obtained by dedifferentiating fibroblasts or epithelial cells in vitro [5]. Somatic stem cells are found in various niches throughout life. Some are multipotent, able to transform into all cells of a certain tissue (e.g., bone marrow progenitor cells). In contrast, others are unipotent and can differentiate into only one type of mature cell (e.g., cells of the basal layer of the epidermis) [6]. Currently, the cells most extensively studied in experimental biology and medicine are mesenchymal (mesodermal) stem cells (MSCs). These cells are found in various mesodermal tissues in the human body, such as the placenta, amniotic fluid, umbilical cord, adipose tissue, testes, and lungs [7].

### 2.2. Regenerative Properties of MSCs

The data on mesenchymal stem cells (MSCs) indicate that they have the potential to differentiate into various types of cells, which can be useful for generating tissue replacements [8,9]. However, limited evidence shows that MSCs utilize this potential in the body [10]. Instead, most research suggests that MSCs promote tissue repair through cells’ interactions and the release of beneficial substances such as growth factors (GFs) and antioxidants [11]. These substances are released in their free form or within small vesicles called exosomes or microvesicles, allowing cellular communication [12]. Furthermore, the unique microRNA patterns within MSCs’ vesicles vary, depending on the studied disease [13]. MSCs can home in on damaged or inflamed areas due to specific receptors and molecules on their surface. Once they reach these areas, they can transfer mitochondria to the damaged cells, which can help restore cellular function and promote healing [14,15,16,17,18,19]. The transfer of mitochondria can occur through various mechanisms such as nanotube tunneling, microvesicles, or cellular fusion [20,21,22].

### 2.3. Immunomodulatory Properties of MSCs

MSCs can regenerate and modulate immune responses. In MSCs, MHC Class I antigens are expressed at a low level, while they do not express MHC Class II antigens or specific co-stimulatory molecules. That means that infusing allogeneic stem cells does not lead to a significant immune response [23]. MSCs possess regenerative capacities and can modulate immune responses, largely due to their secretome, which includes extracellular vesicles and exosomes. While MSCs express low levels of MHC Class I antigens and lack MHC Class II antigens, making them less likely to trigger a significant immune response, their immunomodulatory properties are mediated through the suppression of Th17 lymphocytes, the enhancement of regulatory T-cells, and the promotion of anti-inflammatory cytokines [23,24]. These mechanisms have shown promise in treating autoimmune conditions such as inflammatory joint and intestinal diseases [25,26]. Studies with exogenous MSC infusions have shown that their anti-inflammatory effects are not primarily due to direct interactions with immune cells in the inflamed tissue but rather through their secretome, partly contained within exosomes or microvesicles [27]. Carefully isolated extracellular vesicles of umbilical cord MSCs have been shown to have a strong immunosuppressant effect in vitro, as opposed to other fractions of the MSC-conditioned medium [28].

## 3. Source of MSCs for Research Purposes

MSCs (or, more recently, mesenchymal stromal cells), were discovered in bone marrow by Friedenstein et al. [29,30]. Over subsequent years, MSCs were isolated from different tissues and organs, including the umbilical cord, placenta, peripheral blood, adipose tissue, amniotic fluid, and skeletal muscles [31,32,33,34,35,36,37].

The most frequently used source for MSCs in clinical treatments, including treating kidney diseases, is bone marrow. However, the use of bone marrow-derived MSCs (BM-MSCs) has become limited due to factors such as the risk of viral exposure and the cells’ reduced capability for proliferation/differentiation with increased donor age. Alternative sources, such as MSCs derived from umbilical cord tissue, are being explored, as they exhibit enhanced proliferative potential and reduced immunogenicity [38]. Therefore, researchers have begun exploring other types of MSCs for kidney regeneration. Among the many sources, adipose tissue-derived MSCs (AD-MSCs) and umbilical cord-derived MSCs (UC-MSCs) have become desirable candidates because a large amount of the MSCs can be obtained using relatively minimal invasive procedures [39].

In kidney disease, MSCs are among the most efficient types of cell populations for activating regeneration in a damaged kidney [40]. Pre-clinical reports have demonstrated the therapeutic potential of MSCs in animal models of AKI and CKD [41,42]. A systematic review of more than 70 articles showed that MSCs are among the most effective cell populations for treating experimental CKD [43]. Meanwhile, in a meta-analysis involving animal models of chronic and AKI, MSCs led to kidney regeneration despite the variable modes of administration (arterial, venous, or renal) [41]. Evidence suggests the beneficial effects of MSCs in blocking the AKI–CKD transition, a term used to describe an incomplete recovery from AKI, which results in long-term functional deficits and an increased risk of developing CKD over time [44].

Fat, with its less invasive procurement process and higher concentration of MSCs than bone marrow, is a promising source of MSCs. Fat-derived MSCs also show a lower expression of MHC Class I antigens and have greater replicative and secretory potential [1,45,46]. This potential of fat-derived MSCs is an exciting area of research.

One of the most promising aspects of MSC research is the potential for a non-invasive method of collection, which involves isolating them from urine. In 2008, Zhang et al. from North Carolina identified cells in urine (at a rate of 2–7 cells per 100 mL) that were able to adhere to plastic material and form colonies of differentiated daughter cells. These daughter cells expressed membrane markers characteristic of urothelial, endothelial, and interstitial cells, or myocytes [47,48]. This non-invasive approach adds a practical dimension to the research.

In further research, these cells were successfully differentiated into endodermal, ectodermal, and mesodermal lineages using appropriate culture media [49]. Unlike mesenchymal stem cells (MSCs), up to 75% of urine-derived cells demonstrated telomerase activity, indicating a higher replicative potential without an increased risk of tumorigenesis [50]. These cells are most likely of glomerular origin—MSC-like cells with significant differentiation potential have been isolated from the renal cortical decapsulated glomeruli [51] and have shown nephroprotective effects in renal ischemia–reperfusion injury (IRI) [52]. These cells appear distinct from renal perivascular MSC-like cells, with limited differentiation capabilities (no adipogenesis) but exhibit significant kidney-reparative properties. This was demonstrated in vitro through injury to tubular epithelial cell lines or non-ischemic acute kidney injury (AKI) in mice [53].

### 3.1. Induction of Repair Processes after Acute Kidney Injury

One of the main areas of study for MSCs is their impact on renal ischemia–reperfusion injury, which is the most common cause of acute kidney injury (AKI) in clinical settings, such as shock, cardiac arrest, extracorporeal circulation, and the peritransplantation period. Apart from apoptosis resulting from energy deficiency and acidosis during ischemia, reperfusion leads to additional tissue damage due to oxidative stress and inflammatory reactions. Research conducted thus far has demonstrated that an MSC infusion mitigates kidney IRI. Regardless of the method of MSCs’ administration (either to the renal artery or intravenously, at different timings in relation to IRI), animal models have displayed a less severe course of acute kidney failure [54], along with reductions in oxidative damage and the local expression of inflammatory cytokines [55], an increased renal pool of regulatory T lymphocytes [56], faster regeneration of the renal tubular epithelium [57], and reduced subsequent fibrosis of the renal interstitium [58].

Intravenous infusion of MSCs was equally effective in protecting the kidneys in a model of toxin-induced AKI. In mice, 2 × 10^5^ MSCs, injected 2 h after administration of adriamycin, reduced proteinuria and renal failure present on Day 7 in control mice. This could be the consequence of the inhibition of apoptotic processes and oxidative stress in the tubular cells [59]. These beneficial effects of MSCs are due to their secretory properties, rather than their ability to replicate and differentiate. In an experimental study, rats that underwent 40 min of kidney ischemia were given labeled allogenic bone marrow-derived MSCs (10^6^ cells) through the aorta immediately after or 24 h after renal reperfusion. In both cases, 2 h after the infusion, MSCs were found in the kidney tissue (in peritubular and glomerular capillaries), but were not detected and did not differentiate into other cells during the subsequent 22 and 70 h. Faster normalization of renal excretory function, reduced renal expression of proinflammatory cytokines (Interleukin-1β, tumor necrosis factor α, Interferon γ), and higher renal expression of anti-inflammatory and anti-apoptotic factors, such as Interleukin-10, basic fibroblast GF, TGF α, and Bcl-2, were observed at the end of experiment [60]. Italian researchers have shown that the fraction of the MSC’s secretome responsible for this kidney-protective effect may be largely RNA. Microvesicles isolated from a human bone marrow MSC medium, administered intravenously to rats after 45 min of single-kidney ischemia, reduced acute renal failure and the atrophy of tubular cells. However, subjecting these microvesicles to RNase abolished their beneficial effects in this experimental model [61].

### 3.2. Immunomodulation of Kidney Transplantation

#### 3.2.1. Animal and In Vitro Models

Ischemia–reperfusion injury (IRI) commonly occurs in kidney transplant recipients and can lead to delayed graft function. Mesenchymal stem cell (MSC) therapy has shown promise in this context due to its potential immunosuppressive effects, which could enhance the effectiveness of conventional anti-rejection medications. However, there are different protocols for administering MSCs and animal studies, and limited human observations that support using MSC-based therapies in kidney transplant patients. There is currently no clear preference for the source of MSCs (autologous, donor-derived, or third-party) [1].

In rat models of allogeneic or syngeneic kidney transplantation, infusions of allogeneic bone marrow MSCs into the graft artery during reperfusion reduced the infiltration of CD8+ lymphocytes and monocytes in the organ and mitigated graft rejection [62]. MSCs were also effective when administered intravenously. In rats, a syngeneic MSC infusion during kidney transplantation was found to reduce the expression of inflammatory cytokines in the graft [63].

In mice, MSCs administered intravenously 24 h before kidney transplantation increased the pool of regulatory T-cells in the spleen and prolonged survival of the transplanted kidney (which was not observed with the infusion performed at 24 h post-transplantation) [64].

An experimental study from Germany also found unfavorable effects of MSC infusions in the peritransplant period. Rats were treated with syngeneic or donor-derived bone marrow MSCs intravenously four days before kidney transplantation. They showed symptoms of more severe cellular and humoral rejection and worse graft function on the 10th day after graft implantation [65].

#### 3.2.2. Human Clinical Evidence

In initial studies of human MSCs’ use in renal transplantation, adipose MSCs derived from the perirenal fat of the living kidney donor or the third-party MSCs inhibited the anti-donor and anti-third-party alloreactivity of recipient’s T lymphocytes [66].

This finding was followed by the first clinical studies of using MSCs in living-donor kidney transplant recipients conducted in Italy. In total, two patients underwent intravenous administration of autologous bone marrow MSCs one week after transplantation (1.7 × 10^6^ and 2.0 × 10^6^ cells per kg body weight, respectively), while the other two were given autologous MSCs 24 h before transplantation (2.0 × 10^6^ cells per kg body weight intravenously). Over the five- to seven-year follow-up, the rate of yearly decline in mean renal function was lower by ~70% than in non-MSC-treated transplant patients [67].

It should be stressed that the recipients of MSCs showed considerable variability in their clinical course. One patient developed calcineurin inhibitor-free graft tolerance while the other one experienced acute graft rejection two weeks after transplantation, both of them being ones that were given MSCs before kidney implantation. Nevertheless, infections or neoplasms were not more frequent in the MSC-treated subjects. Except in one patient, a ~50% decrease in the percentage of memory CD8+ T-cells was observed at one year post-transplantation compared with the pre-transplant levels, which was not seen in any of the controls [67].

In the largest clinical trial conducted, 105 Chinese renal transplant recipients were administered autologous MSCs at graft reperfusion and again after two weeks in place of anti-IL-2 receptor antibodies. Such induction was associated with more rapid organ regeneration over the first month post-transplantation. Additionally, a lower rate of cellular rejection (7.6% vs. 21.6% in the control group) was recorded, with a milder course in the six-month follow-up [68].

On the other hand, studies appeared that denied the beneficial effects of intravenous infusions of MSCs on the outcome of kidney transplant; improvements in the function of renal allograft and rats’ survival were found only when allogeneic fat MSCs were injected into the graft artery, and not when they were administered intravenously at implantation [68].

In the evolving field of stem cell therapies for kidney transplantation, research has expanded beyond mesenchymal stem cells (MSCs) to include other types of stem cells, such as hematopoietic stem cells (HSCs). A notable study by Leventhal et al. investigated the use of HSCs in inducing tolerance among kidney transplant recipients. This study involved the administration of HLA-mismatched kidneys along with tolerogenic graft-facilitating cells (FCs) and HSCs, following a conditioning regimen with fludarabine, total body irradiation, and cyclophosphamide. Post-transplant immunosuppression was managed with tacrolimus and mycophenolate mofetil [69]. The results showed transient chimerism in most patients, with some achieving persistent chimerism and donor-specific tolerance, allowing them to discontinue immunosuppression after one year. However, complications such as viral sepsis and renal artery thrombosis were reported. This highlights the potential of HSCs to contribute to long-term tolerance in kidney transplantation but also underscores the associated risks, including infection-related complications, that need to be addressed. Integrating HSC-based therapies into our discussion underscores the diversity of approaches in stem cell research for kidney transplantation and the need for ongoing investigation to optimize these strategies for clinical use.

Even more discouraging are the recent findings of another Chinese team of researchers, who injected allogeneic umbilical cord blood MSCs into 21 recipients intravenously immediately prior to transplantation (2 × 10^6^/kg body weight) and, additionally, into the graft artery at reperfusion (5 × 10^6^), in addition to standard immunosuppression. In the one-year follow-up period, no statistically significant differences were found from the controls regarding postoperative and infectious complications, renal function, the frequency of rejection, or the survival time of the kidney transplant [70].

This table provides an overview of key findings from various studies on the use of mesenchymal stem cells (MSCs) in kidney transplantation. It includes information on the source and administration methods of MSCs, the main outcomes observed in both animal models and human clinical trials, and their effects on kidney transplant outcomes. The Table 1 highlights differences in administration protocols for MSCs, the impact on graft function and survival, and notable variations in clinical responses among patients. This summary aims to offer a concise comparison of the effectiveness and challenges associated with MSC therapies in the context of kidney transplantation.

#### 3.2.3. Clonal Heterogeneity of MSC Cultures

Another important aspect to consider in the context of MSC-based therapies is the clonal heterogeneity of MSC cultures. This heterogeneity can partially explain the significant variability in clinical outcomes observed among patients who have received MSCs for modulation of transplantation-related immune tolerance.

Mesenchymal stem cells (MSCs) are known to exhibit substantial clonal heterogeneity, meaning that even within a single MSC culture, there can be a diverse range of cell subpopulations with differing properties and functions. This variability can result from differences in the cells’ origin, the passage number, and the microenvironment in which the MSCs are expanded [72,73].

The presence of different MSC subpopulations within a culture can lead to variability in the therapeutic outcomes. Some MSCs might have stronger immunomodulatory capabilities, while others may not be as effective, leading to inconsistent results across clinical trials. This clonal heterogeneity can impact the efficacy of MSC-based therapies in modulating immune tolerance and influencing transplant outcomes [74].

To address this issue, researchers and clinicians should consider the following:

(1) Standardization of MSC cultures: Developing standardized protocols for the isolation, expansion, and characterization of MSCs can help minimize clonal heterogeneity and improve consistency in clinical results [75].
(2) Characterization of MSC subpopulations: Employing advanced techniques to identify and quantify different MSC subpopulations within a culture can provide insights into their functional capabilities and potential therapeutic efficacy [76].
(3) Personalized approaches: Tailoring MSC therapies to individual patients based on their specific immune profiles and MSC characteristics may enhance the effectiveness and predictability of treatment outcomes [77].

**Figure 1:**
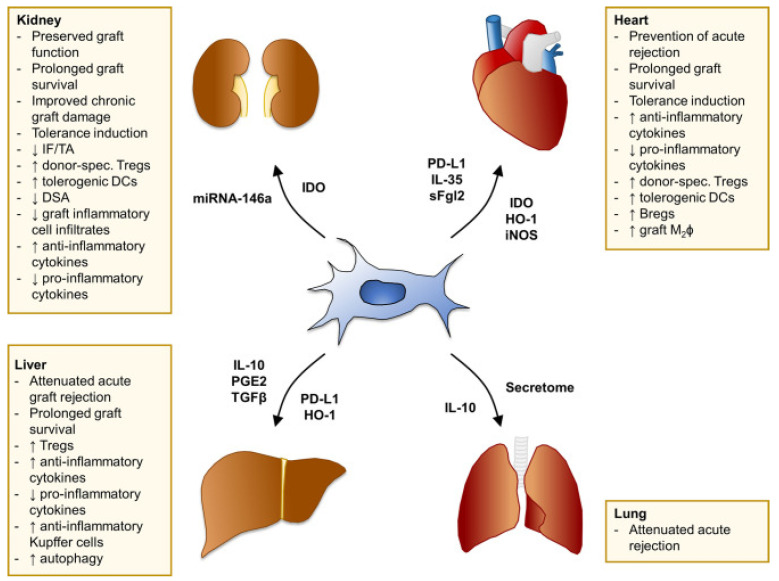
Summary of MSC effects in pre-clinical models of solid organ transplantation. Main findings of studies with MSC in experimental models of kidney, heart, liver, and lung transplantation. The mediators involved in MSC-induced pro-tolerogenic effects and/or overexpressed explicitly in selected MSC cell lines through genetic engineering are listed next to each arrow. Bregs, regulatory B cells; DCs, dendritic cells; DSA, donor-specific antibodies; HO-1, heme oxygenase-1; IDO, indoleamine 2,3-dioxygenase; IF/TA, interstitial fibrosis/tubular atrophy; IL-, interleukin-; iNOS, inducible nitric oxide synthase; M_2_ϕ, M2 macrophages; PD-L1, programmed death-ligand 1; PGE2, prostaglandin E2; sFgl2, soluble fibrinogen-like protein 2; TGFβ, transforming growth factor β; Tregs, regulatory T cells.

By addressing clonal heterogeneity, we can better understand and potentially mitigate the variability in clinical outcomes associated with MSC therapies, ultimately improving their application in transplantation and other therapeutic areas [Figure 1].

## 4. Mesenchymal Stem Cells and Transplant Tolerance

Mesenchymal stem cells (MSCs) possess exceptional capabilities that render them highly valuable in various fields of medicine, including transplant immunology.

The unique combination of plasticity and non-immunogenicity makes MSCs promising candidates for various therapeutic applications, particularly in transplant immunology, where their ability to modulate immune responses can potentially improve outcomes in transplantation and reduce the need for immunosuppressive drugs [78].

MSCs must meet specific criteria, including adherence to plastic material under standard culture conditions; expression of CD105, CD73, and CD90 markers; and the absence of CD34, CD45, CD11a, CD19, CD79a, CD14, CD11b, and histocompatibility locus antigen (HLA)-DR. Additionally, they must demonstrate the ability to differentiate into osteocytes and adipocytes in response to specific stimuli [79,80].

### Experimental Studies

Le Blanc et al. [81] obtained bone marrow from healthy human volunteers and cultured mesenchymal stem cells (MSCs). MSCs isolated from the second or third passages were then co-cultured with peripheral blood lymphocytes in different ratios, and they found that MSCs showed diverse responses, including inhibition of T-lymphocytes’ proliferation and sometimes even stimulation of DNA synthesis.

One significant mechanism of action for MSCs involves the secretion of HLA-G5, which plays a crucial role in suppressing T-cells and NK cells, shifting the T-cells’ response towards T-helper Type 2 (Th2), and promoting the generation of T-regulatory cells (CD4+ CD25hi forkhead box P3 (FoxP3+) [81,82,83].

Rodent models are commonly used in biomedical research to study various diseases, conditions, and treatments before moving to human trials. In the context of transplantation and MSC research, rodent models (such as mice or rats) are used to mimic aspects of human physiology and immune responses. Rodent models offer a controlled environment in which to study the effects of MSCs on the outcomes of transplantation. Specifically, in the study by Casiraghi et al. [71,84], they investigated the timing and dosage effects of MSCs in a rodent transplantation model. Their study revealed that administering autologous MSCs post-transplantation in murine models resulted in increased neutrophil infiltration and the deposition of complement in the renal allograft, ultimately leading to rejection.

Conversely, when MSCs were administered before transplantation, they targeted lymphoid organs, which improved graft survival and promoted the generation of T-regulatory cells. Therefore, these investigations collectively highlighted the promising potential of MSCs in fostering transplant tolerance prior to solid organ transplantation.

After previous failures in achieving tolerance, researchers renewed their interest in stem cell therapy when Scandling et al. infused donor HSCs in 12 patients who underwent HLA-matched kidney transplantation under a non-myeloablative conditioning regimen [71]. Ten days after kidney transplantation, these patients underwent a conditioning regimen that included 10 doses of TLI (80 to 120 cGy) targeting the lymph nodes, spleen, and thymus, along with five doses of rabbit anti-thymocyte globulin. On Day 11, CD34+ selected cells from the donors (ranging from 5 × 10^6^ to 16 × 10^6^/kg) and a defined dose of T-cells (ranging from 1 × 10^6^ to 10 × 10^6^ per kilogram) were intravenously injected at the outpatient infusion center. All patients received mycophenolate mofetil for one month and cyclosporine, starting at Day 0 for at least six months. Cyclosporine was discontinued 6 to 17 months after transplantation as long as chimerism persisted for at least 6 months, with no evidence of graft-versus-host disease, clinical rejection, or surveillance biopsy-proven rejection at the time of withdrawal. In the study by Le Blanc et al. [85], the hematopoietic stem cells were modified through non-myeloablative conditioning and co-infusion with donor-derived mesenchymal stem cells to promote tolerance. This combination aimed to reduce the risk of graft-versus-host disease while encouraging the development of stable mixed chimerism, thus facilitating the discontinuation of immunosuppressive therapy. 

They reported success in 8 out of 12 patients and conducted a mean follow-up of 25 months. However, they noted a recurrence of focal segmental glomerulosclerosis (FSGS) in one patient. This conditioning can be lethal to patients, especially in developing countries, where infection risks are higher and markers of immune tolerance, as well as regular monitoring, are not clearly addressed. Another important fact to mention is that recipient–donor HLA matching is mandatory, which may only be clinically feasible sometimes. 

In research conducted by Leventhal et al. [69], they attempted to induce tolerance in eight kidney transplant recipients using hematopoietic stem cells (HSCs) within a conditioning regimen. Key aspects of this study include the administration of HLA-mismatched kidneys and tolerogenic graft facilitating cells (FCs), alongside HSCs, following conditioning with fludarabine, 200-centigray total body irradiation, and cyclophosphamide. Post-transplant immunosuppression was maintained with tacrolimus and mycophenolate mofetil [69]. The lowest absolute neutrophil counts were observed approximately one week after transplantation, followed by recovery within two weeks. Multilineage chimerism ranged from 6% to 100% in their patients at one month post-transplant. The conditioning regimen was well tolerated, and the patients were managed on an outpatient basis, starting from postoperative Day 2. Cyclosporine was discontinued 6 to 17 months after transplantation as long as chimerism persisted for at least 6 months, with no evidence of graft-versus-host disease, clinical rejection, or surveillance biopsy-proven rejection at the time of withdrawal. In the study by Le Blanc [85], the hematopoietic stem cells were modified through non-myeloablative conditioning and co-infusion with donor-derived mesenchymal stem cells to promote tolerance. This combination aimed to reduce the risk of graft-versus-host disease while encouraging the development of stable mixed chimerism, thus facilitating the discontinuation of immunosuppressive therapy.

The complications following transplantation in this case included transient chimerism (treated with low-dose tacrolimus), viral sepsis two months after transplant, and renal artery thrombosis.

Five subjects maintained persistent chimerism, exhibited immunocompetence, and showed donor-specific tolerance through in vitro proliferative assays. They successfully discontinued all immunosuppression one year after transplantation. None of the recipients produced anti-donor antibodies, exhibited engraftment syndrome, or developed graft-versus-host disease. The authors concluded that modifying a mobilized stem cell graft and using non-myeloablative conditioning presents a safe, practical, and reliable method for achieving persistent chimerism and donor-specific tolerance in recipients of solid organ transplants. The application of therapy involving the manipulation of stem cell grafts and non-myeloablative conditioning may increase the risk of infection-related complications, making this strategy less safe and less encouraging in dialysis settings.

Tan et al. [68] conducted a study involving autologous bone marrow-derived MSCs in 105 renal transplant (RT) patients. They administered MSCs twice: before the anastomosis and two weeks after the renal transplant (RT), and they found that bone marrow-derived MSCs were safe and led to improved renal function, along with a reduced incidence of infections during one year of follow-up [81].

Ongoing trials across all continents are investigating the use of bone marrow-derived MSCs to mitigate tissue injury in autoimmune disorders and enhance the long-term success of transplants.

Perico et al. [86] administered autologous MSCs seven days after renal transplantation in two recipients who received living-related kidneys. These patients underwent T-cell depletion therapy and continued to take cyclosporine and mycophenolate mofetil as maintenance immunosuppressive therapy. They were monitored for approximately one year after the procedure, and at the one-year mark, both patients showed increases in T-regulatory cells (CD4+CD25high FoxP3+ CD127−), along with a decrease in CD8+ cells and stable graft function.

A study by Aruna et al. [87] investigated 606 living donor kidney transplants. They aimed to eliminate rejecting T- and B-cells using non-myeloablative conditioning, which included total lymphoid irradiation (200 cGy administered over 4 or 5 days), bortezomib (1.5 mg/kg body weight in four divided doses every third day), cyclophosphamide (20 mg/kg body weight), and rabbit anti-thymocyte globulin (1.5 mg/kg body weight). They infused a combination of MSCs derived from adipose tissue and HSCs into the portal and thymic circulation, taking advantage of the liver’s high tolerance due to its unique microanatomy and diverse functional characteristics. Cells entering the thymus undergo processes of positive and negative selection, which leads to the development of T-cells that can respond to a wide array of foreign antigens while avoiding reactivity against self-antigens. The thymus produces a specific type of regulatory T-cells that suppress the self-reactivity of T-cell clones that might evade negative selection. Therefore, the thymus is considered crucial for promoting tolerance [87].

Several investigations have examined bone marrow-derived MSCs (BM-MSCs) and adipose tissue-derived MSCs (AT-MSCs), often with small donor groups and varying patient ages. These studies indicated that both types of MSCs may suppress the proliferation of peripheral blood mononuclear cells (PBMCs), yet they observed no notable differences between the two MSC populations [88,89,90]. Research conducted by Sara M. Melief et al. [46] suggested that adipose tissue-derived multipotent stromal cells (AT-MSCs) might be a more effective alternative for immunomodulatory treatment compared with bone marrow-derived multipotent stromal cells (BM-MSCs). The research compared the immunomodulatory abilities of BM-MSCs and AT-MSCs from age-matched donors, revealing that both cell types possess a similar immunophenotype and in vitro multilineage differentiation potential. Although both BM-MSCs and AT-MSCs are used to suppress the proliferation of activated peripheral blood mononuclear cells and inhibit the differentiation of monocyte-derived immature dendritic cells, AT-MSCs displayed stronger immunomodulatory effects at equivalent cell numbers [91]. This enhanced effect is due to the increased secretion of crucial cytokines such as Interleukin-6 and transforming growth factor-β1, along with the higher metabolic activity of AT-MSCs. As a result, fewer AT-MSCs are needed to achieve the same level of immunomodulation, suggesting their potential as a superior option for immunomodulatory therapy [92].

The overall explanation for the superior functionality of AT-MSCs is likely their heightened metabolic activity, which leads to the production of greater levels of cytokines involved in the immunosuppressive mechanisms of MSCs. This applies to other factors in MSC-mediated immunomodulation as well, such as PGE2, galectin-1, and HLA-G5 [93].

In summary, AT-MSCs (adipose tissue-derived multipotent stromal cells) have been shown to be superior to BM-MSCs (bone marrow-derived multipotent stromal cells) due to several key factors as follows.

1. Higher metabolic activity: AT-MSCs have greater metabolic activity, meaning they can produce more energy and biological molecules that are necessary for their functions.
2. Increased cytokine production: cytokines are proteins with a cardinal role in the regulation of the immune response. AT-MSCs produce higher amounts of cytokines such as Interleukin-6 and transforming growth factor-β1, which are important for immunosuppressive mechanisms.
3. Other immunomodulatory factors: in addition to cytokines, AT-MSCs produce other molecules such as PGE2 (prostaglandin E2), galectin-1, and HLA-G5, which contribute to their ability to modulate the immune response.

All of these together mean that AT-MSCs can be more effective at suppressing undesirable immune reactions, making them potentially a better choice for therapies that require immunomodulation [93,94].

Studies performed on animal and human models have demonstrated that MSCs derived from bone marrow are safe and feasible for treating autoimmune disorders and protecting grafts from injury during transplantation, leading to improved long-term outcomes. Some unresolved issues include identifying the most suitable source of MSCs, determining the optimal timing and dosage for infusion, selecting the infusion site, and assessing the effectiveness of recovery and/or the reduction in immunosuppression.

## 5. Discussion

Kidney transplantation (KT) continues to be the main therapeutic approach for end-stage renal disease (ESRD). Nonetheless, the widening gap between the availability of organs and patients’ needs has led to more inclusive donor criteria. This shift has resulted in an increase in the use of marginal kidneys for transplantation in high-risk patients. This proactive strategy has notably increased the frequency of delayed graft function (DGF), which is characterized by the requirement for dialysis within the first week after transplantation [95]. DGF is a complex condition that negatively impacts the survival of both the patient and the graft [96]. Major risk factors for DGF encompass expanded criteria donors (ECD), donation after circulatory death (DCD), extended warm ischemia time (WIT) or cold ischemia time (CIT), and sensitization of the recipient [97].

Considering the different peritransplant events that contribute to the development of DGF, ischemia–reperfusion injury (IRI) deserves special attention, as it is unavoidable and a major factor in acute tubular necrosis (ATN), the primary histological finding associated with DGF [98]. IRI is associated with a substantial proinflammatory response that can initiate various cell death pathways, lead to endothelial dysfunction, cause transcriptional reprogramming, and activate both the innate and adaptive immune systems [94]. Due to the strong correlations of IRI, DGF, acute rejection (AR), and progressive interstitial fibrosis with tubular atrophy (IF/TA), as well as their detrimental impact on kidney allografts’ function and survival, the prevention and treatment of IRI have become primary concerns for the transplant community [99].

Growing evidence suggests that adipose stem/stromal cells (ASCs) have unique traits that might aid in preventing, mitigating, or reversing IRI. Additionally, their immunomodulatory and tolerogenic properties have led to the exploration of ASC-based preventive and therapeutic approaches in both pre-clinical and clinical models of renal IRI and allograft rejection. ASCs are plentiful, easily obtainable, and can be readily expanded in culture. Moreover, ASCs have the capability to release extracellular vesicles (EVs), which might serve as effective agents for tissue repair and promoting tolerance. This review examines the current understanding of how ASCs and ASC-derived EVs function and the therapeutic potential they offer in the context of kidney transplantation.

The most relevant pre-clinical and clinical studies, as well as actual limitations and future perspectives, are highlighted.

Key findings from the studies highlight several important aspects, as follows.

1. Safety and feasibility: most studies have confirmed the safety of the application of MSCs in the context of transplantation, with minimal reported adverse effects or complications associated with the therapy. This is crucial for further advancing the clinical applications.
2. Impact on immunosuppression: some studies have suggested that MSCs may reduce the need for immunosuppressive therapy post-transplantation. This is significant, as it could improve the long-term outcomes of transplanted organs and reduce the risks associated with immunosuppression.
3. Need for further research: despite positive findings, there are still open questions, such as the optimal dosage, timing, and method of administration of MSCs. Further research is needed to understand these aspects better and define the best clinical practices.
4. Perspectives on transplant tolerance: studies exploring the combination of MSCs and hematopoietic stem cells (HSCs) as a means to induce transplant tolerance present particularly promising results. This approach can shift the standard paradigm in transplantation medicine towards strategies promoting immune tolerance.
5. Challenges and opportunities: despite progress, challenges such as the need for individualized approaches for each patient and the requirement for further studies to confirm the long-term clinical benefits of MSCs’ application.
6. Impact of socioeconomic factors: infections remain a major challenge for all transplantations, especially in developing countries, where the social, economic, and environmental conditions do not support optimal health outcomes. In developing countries, infections such as tuberculosis, cytomegalovirus, and bacterial infections significantly affect post-transplant outcomes. The financial burden of transplantation, coupled with limited access to healthcare and lack of insurance coverage for dialysis after graft failure, exacerbates the economic hardships faced by patients and their families. Research on transplant tolerance using MSCs holds promise for improving outcomes in these vulnerable populations by potentially reducing the need for lifelong immunosuppressive medications and the associated costs.
7. Economic benefits: The use of MSCs, including adipose tissue-derived MSCs (AD-MSCs), has shown promise in reducing the overall cost burden of transplantation. For instance, in Ahmedabad, India, the total cost of transplantation using AD-MSCs was approximately USD 6000, significantly lower than traditional transplantation costs. This reduction lowered the financial strain on patients and decreased the monthly healthcare costs from approximately USD 2000 to less than USD 50. Additionally, minimizing infections due to reduced immunosuppressive requirements allows patients to return to work and have a normal life, improving overall quality of life post-transplant.

### 5.1. Limitations of MSC-Based Therapies

While mesenchymal stem cell (MSC) therapies offer promising potential in kidney transplantation, several limitations and concerns must be addressed to fully understand their efficacy and safety. One significant issue is the potential activation of the complement system during the infusion of MSCs [100,101].

### 5.2. Complement Activation

The complement system, a key component of the innate immune response, can be activated during the infusion of MSCs. Complement activation can lead to inflammation and tissue damage, which may counteract the therapeutic benefits of MSCs. Several studies have indicated that the infusion of MSCs might trigger complement activation, potentially leading to adverse effects such as an increased risk of graft rejection or impaired graft function [102,103,104].

In clinical settings, complement activation during the infusion of MSCs has been observed in some patients, leading to concerns about the safety and efficacy of these therapies. Monitoring markers of complement activation and implementing strategies to mitigate this activation are crucial for improving patient outcomes and optimizing MSC-based therapies.

Future studies should focus on the following.

(1) Assessing the extent of complement activation in different MSC infusion protocols [105].
(2) Developing strategies to minimize complement activation, such as using complement inhibitors or optimizing the preparation methods of MSCs [106].
(3) Evaluating the impact of complement activation on long-term transplant outcomes and patients’ safety [107].
(4) By addressing these limitations, researchers can better harness the potential of MSC therapies and enhance their application in kidney transplantation.

In conclusion, MSCs have a promising role in the induction and sustenance of transplant tolerance when infused into the liver and thymic circulation pre-transplant. Further experimental and clinical trials are urgently needed to fully explore their potential benefits and refine the protocols for widespread clinical implementation.

## Figures and Tables

**Table 1:** Summary of findings on MSC therapy in kidney transplantation from animal and human studies.

Study Type	MSC Source/Administration	Key Findings	Effect on Kidney Transplant	Reference
Animal Models	Alogenic bone marrow MSCs infused during reperfusion	Reduced CD8+ lymphocite and monocyte infiltration; mitigated graft rejection	Improved graft survival	[63]
Animal Models	Syngeneic MSCs infused during transplantation	Reduced inflammatory cytokine expression in the graft	Enhanced graft function	[63]
Animal Models	Intravenous MSCs administered 24 h before transplantation	Increased regulatory T-cells prolonged graft survival	Improved kidney survival	[71]
Animal Models	Syngeneic or donor-derived MSCs administered intravenously four days before transplantation	Severe cellular and humoral rejection; worse graft function	Deteriorated graft function	[65]
Human Clinical	Adipose MSCs from living donor or third-party	Inhibited anti-donor and anti-third party T lymphocite reactivity	Potentialy improved renal function; variable patients outcomes	[66]
Human Clinical	Autologous bone marrow MSCs administered one week or 24 h before transplantation	Reduced annual decline in renal function;variable patient outcomes	Improved renal function; some cases of graft tolerance	[68]
Human Clinical	Autologous MSCs administered at graft reperfusion and after two weeks	Faster organ regeneration;lower rate of cellular rejection	Enhanced graft survival and function	[67]
Human Clinical	Intravenous of graft artery administration of allogenic fat MSCs	No significant improvement in graft function compared to controls	No improved outcomes	[68]
HSC Therapy	HLA-mismatched kidneys with HSCs and FCs	Transient chimerism; some achieved donor-specific tolerance	Potential for long-term tolerance risk of complications	[67]
Human Clinical	Allogenic umbilical cord blood MSCs intravenously and in graft artery	No significant diferences in outcomes compared to controls	No impared outcomes	[67]
